# Supplementary material for: Unveiling the Bioactive Potential of the Invasive Jellyfish Phyllorhiza punctata Through Integrative Transcriptomic and Proteomic Analyses
Source: Biomolecules. 2025 Aug 4;15(8):1121. doi: 10.3390/biom15081121 (PMC12383608; doi:10.3390/biom15081121)
Supplement: Supplementary file 1 [file biomolecules-15-01121-s001.zip › Suplementary Material/FileS3/PEPFOLD4/XP_065052365_12919/clusters.html]

 PEP-FOLD clusters


Download archive of all models  here   

|  |  |  |
| --- | --- | --- |
| # | file | sOPEP |
| # cluster 1 - Size: 1 |  |  |
|  | XP\_065052365\_12919-model1 | -23.6635 |
| # cluster 2 - Size: 1 |  |  |
|  | XP\_065052365\_12919-model2 | -21.056 |
| # cluster 3 - Size: 1 |  |  |
|  | XP\_065052365\_12919-model3 | -16.4966 |
| # cluster 4 - Size: 1 |  |  |
|  | XP\_065052365\_12919-model4 | -13.5329 |
| # cluster 5 - Size: 1 |  |  |
|  | XP\_065052365\_12919-model5 | -12.9986 |
| # cluster 6 - Size: 1 |  |  |
|  | XP\_065052365\_12919-model6 | -12.6592 |
| # cluster 7 - Size: 4 |  |  |
|  | XP\_065052365\_12919-model7 | -12.594 |
|  | XP\_065052365\_12919-model7.1 | -12.5202 |
|  | XP\_065052365\_12919-model7.2 | -12.3456 |
|  | XP\_065052365\_12919-model7.3 | -12.199 |
| # cluster 8 - Size: 20 |  |  |
|  | XP\_065052365\_12919-model8 | -12.0754 |
|  | XP\_065052365\_12919-model8.1 | -11.8681 |
|  | XP\_065052365\_12919-model8.2 | -11.578 |
|  | XP\_065052365\_12919-model8.3 | -11.3232 |
|  | XP\_065052365\_12919-model8.4 | -11.2244 |
|  | XP\_065052365\_12919-model8.5 | -11.1949 |
|  | XP\_065052365\_12919-model8.6 | -10.9163 |
|  | XP\_065052365\_12919-model8.7 | -10.8792 |
|  | XP\_065052365\_12919-model8.8 | -10.4505 |
|  | XP\_065052365\_12919-model8.9 | -10.2148 |
|  | XP\_065052365\_12919-model8.10 | -10.1639 |
|  | XP\_065052365\_12919-model8.11 | -10.0459 |
|  | XP\_065052365\_12919-model8.12 | -9.93637 |
|  | XP\_065052365\_12919-model8.13 | -9.92705 |
|  | XP\_065052365\_12919-model8.14 | -9.8631 |
|  | XP\_065052365\_12919-model8.15 | -9.60283 |
|  | XP\_065052365\_12919-model8.16 | -9.57829 |
|  | XP\_065052365\_12919-model8.17 | -9.55735 |
|  | XP\_065052365\_12919-model8.18 | -9.5308 |
|  | XP\_065052365\_12919-model8.19 | -9.13333 |
| # cluster 9 - Size: 2 |  |  |
|  | XP\_065052365\_12919-model9 | -11.8194 |
|  | XP\_065052365\_12919-model9.1 | -11.4994 |
| # cluster 10 - Size: 8 |  |  |
|  | XP\_065052365\_12919-model10 | -11.7872 |
|  | XP\_065052365\_12919-model10.1 | -11.591 |
|  | XP\_065052365\_12919-model10.2 | -11.4062 |
|  | XP\_065052365\_12919-model10.3 | -11.0355 |
|  | XP\_065052365\_12919-model10.4 | -10.6671 |
|  | XP\_065052365\_12919-model10.5 | -9.73541 |
|  | XP\_065052365\_12919-model10.6 | -9.62276 |
|  | XP\_065052365\_12919-model10.7 | -9.05736 |
| # cluster 11 - Size: 12 |  |  |
|  | XP\_065052365\_12919-model11 | -11.7861 |
|  | XP\_065052365\_12919-model11.1 | -11.645 |
|  | XP\_065052365\_12919-model11.2 | -11.1074 |
|  | XP\_065052365\_12919-model11.3 | -11.0745 |
|  | XP\_065052365\_12919-model11.4 | -10.8505 |
|  | XP\_065052365\_12919-model11.5 | -10.6951 |
|  | XP\_065052365\_12919-model11.6 | -10.323 |
|  | XP\_065052365\_12919-model11.7 | -10.152 |
|  | XP\_065052365\_12919-model11.8 | -9.65367 |
|  | XP\_065052365\_12919-model11.9 | -9.15405 |
|  | XP\_065052365\_12919-model11.10 | -8.87869 |
|  | XP\_065052365\_12919-model11.11 | -7.90698 |
| # cluster 12 - Size: 3 |  |  |
|  | XP\_065052365\_12919-model12 | -11.767 |
|  | XP\_065052365\_12919-model12.1 | -11.6564 |
|  | XP\_065052365\_12919-model12.2 | -11.5772 |
| # cluster 13 - Size: 4 |  |  |
|  | XP\_065052365\_12919-model13 | -11.3236 |
|  | XP\_065052365\_12919-model13.1 | -11.0855 |
|  | XP\_065052365\_12919-model13.2 | -11.07 |
|  | XP\_065052365\_12919-model13.3 | -10.6743 |
| # cluster 14 - Size: 4 |  |  |
|  | XP\_065052365\_12919-model14 | -11.0189 |
|  | XP\_065052365\_12919-model14.1 | -10.4901 |
|  | XP\_065052365\_12919-model14.2 | -10.3612 |
|  | XP\_065052365\_12919-model14.3 | -10.0758 |
| # cluster 15 - Size: 6 |  |  |
|  | XP\_065052365\_12919-model15 | -10.6631 |
|  | XP\_065052365\_12919-model15.1 | -10.098 |
|  | XP\_065052365\_12919-model15.2 | -9.80856 |
|  | XP\_065052365\_12919-model15.3 | -9.74409 |
|  | XP\_065052365\_12919-model15.4 | -8.49867 |
|  | XP\_065052365\_12919-model15.5 | -7.09677 |
| # cluster 16 - Size: 2 |  |  |
|  | XP\_065052365\_12919-model16 | -10.5349 |
|  | XP\_065052365\_12919-model16.1 | -7.67768 |
| # cluster 17 - Size: 4 |  |  |
|  | XP\_065052365\_12919-model17 | -10.0735 |
|  | XP\_065052365\_12919-model17.1 | -9.76949 |
|  | XP\_065052365\_12919-model17.2 | -9.73785 |
|  | XP\_065052365\_12919-model17.3 | -9.25753 |
| # cluster 18 - Size: 1 |  |  |
|  | XP\_065052365\_12919-model18 | -10.0227 |
| # cluster 19 - Size: 6 |  |  |
|  | XP\_065052365\_12919-model19 | -9.80708 |
|  | XP\_065052365\_12919-model19.1 | -9.8046 |
|  | XP\_065052365\_12919-model19.2 | -9.43398 |
|  | XP\_065052365\_12919-model19.3 | -9.39958 |
|  | XP\_065052365\_12919-model19.4 | -9.02337 |
|  | XP\_065052365\_12919-model19.5 | -6.90485 |
| # cluster 20 - Size: 6 |  |  |
|  | XP\_065052365\_12919-model20 | -9.74848 |
|  | XP\_065052365\_12919-model20.1 | -9.70412 |
|  | XP\_065052365\_12919-model20.2 | -9.63826 |
|  | XP\_065052365\_12919-model20.3 | -9.41149 |
|  | XP\_065052365\_12919-model20.4 | -9.16006 |
|  | XP\_065052365\_12919-model20.5 | -8.98196 |
| # cluster 21 - Size: 2 |  |  |
|  | XP\_065052365\_12919-model21 | -9.59461 |
|  | XP\_065052365\_12919-model21.1 | -8.61887 |
| # cluster 22 - Size: 1 |  |  |
|  | XP\_065052365\_12919-model22 | -7.75097 |
| # cluster 23 - Size: 1 |  |  |
|  | XP\_065052365\_12919-model23 | -7.37221 |
| # cluster 24 - Size: 1 |  |  |
|  | XP\_065052365\_12919-model24 | -7.36131 |
| # cluster 25 - Size: 2 |  |  |
|  | XP\_065052365\_12919-model25 | -7.14925 |
|  | XP\_065052365\_12919-model25.1 | -6.79732 |
| # cluster 26 - Size: 1 |  |  |
|  | XP\_065052365\_12919-model26 | -7.05205 |
| # cluster 27 - Size: 1 |  |  |
|  | XP\_065052365\_12919-model27 | -6.95321 |
| # cluster 28 - Size: 1 |  |  |
|  | XP\_065052365\_12919-model28 | -6.74369 |
| # cluster 29 - Size: 1 |  |  |
|  | XP\_065052365\_12919-model29 | -6.73599 |
| # cluster 30 - Size: 1 |  |  |
|  | XP\_065052365\_12919-model30 | -6.65395 |
|  |  |  |
